# How Are Forcibly Displaced People Affected by the COVID-19 Pandemic Outbreak? Evidence From Brazil

**DOI:** 10.1177/00027642211000402

**Published:** 2021-09

**Authors:** Patrícia Nabuco Martuscelli

**Affiliations:** 1 University College London, England, UK

**Keywords:** refugees, Brazil, COVID-19, pandemic, forced migration

## Abstract

Refugees tend to be a neglected population during health emergencies. This article studies how the COVID-19 pandemic outbreak in Brazil affected forcibly displaced people considering their intersectional multiple identities. I conducted 29 semistructured phenomenological interviews with refugees living in the states of São Paulo and Rio de Janeiro between March 27 and April 06, 2020. These states’ governors closed nonessential services and schools. The results indicate that refugees face three challenges connected to this pandemic: (a) same challenges as Brazilians due to their labor vulnerability social identity, (b) challenges aggravated by the pandemic due to their identity of nonnationals including access to information and services, and (c) new challenges due to their social identity of forced displaced nonnationals including closing of migration services and borders and the feeling of “living the pandemic twice.” This research contributes to the literature of intersectionality and asylum by understanding how refugees in the Global South are affected by pandemics and responses to them, considering their own lived experiences and multiple social identities.

As of January 16, 2021, the severe acute respiratory syndrome coronavirus 2 infected 92,262,621 and killed 1,995,037 people worldwide, according to data from the World Health Organization (WHO, 2021). The organization declared the coronavirus disease 2019 (COVID-19) a pandemic on March 11, 2020. Brazil was one of the most affected countries by this pandemic.^1^ Responses to deal with this global health crisis included the closing of borders, nonessential businesses and schools, lockdowns, quarantine, isolation, social distancing, and social and economic programs to answer to a disease with no cure nor approved vaccine accessible worldwide till the date of writing this article. The closures and lockdowns that resulted from COVID-19 affected practically all human beings in the world, including near 272 million people living outside their countries of origin as migrants, or 3.5% of the world population (International Organization for Migration [IOM], 2019). The United Nations High Commissioner for Refugees (UNHCR, 2020a) calculated 26 million refugees at the end of 2019, that is, people who fled their countries of origin due to a well-founded fear of persecution because of their race, nationality, religion, political opinion, or membership in a specific social group (UNHCR, 2020a).

Migrants and refugees are generally left behind in global health discussions (Ventura, 2015). Moreover, they are a neglected, left-behind group in national responses to the pandemic (Raju & Ayeb-Karlsson, 2020). Therefore, this article discusses how the COVID-19 pandemic outbreak in Brazil affects people recognized as refugees according to the Brazilian law (Law 9474/1997). Refugees are a forcibly displaced population that is protected by international law, including the Convention Relating to the Status of the Refugees (1951) and its Protocol (1967). They are a hard to reach population that are vulnerable due to the intersectionality of their vulnerabilities (class, race, being forced displaced). Refugees had a bigger chance to have their rights violated even before this pandemic, but the current situation has aggravated previous vulnerabilities and created new challenges for them.

This pandemic “is a social event that is disrupting our social order” (Teti et al., 2020, p. 1) which demands collective responses. Therefore, it is important to understand how different groups are affected by this virus according to their contextual vulnerabilities (Teti et al., 2020). This article contributes to understanding how refugees themselves perceive the impact of the COVID-19 pandemic outbreak on their lives. UNHCR (2020a) shows that 85% of all refugees in the world live in developing countries. Latin America is currently the epicenter of the pandemic (WHO, 2021), and it is facing an increasingly forced displacement due to the situation in Venezuela. There are many violations of human rights in the country, including persecutions of oppositions to the current government and shortages of medicines, food, and other essential items originating a humanitarian emergency (Human Rights Watch, 2020). In 2019, there were more than 3.6 million Venezuelans displaced abroad (UNHCR, 2020a).

Brazil is an interesting case study. The country has a progressive legislation on asylum with a broad definition of refugees (Jatobá & Martuscelli, 2018). It was the sixth country in the world that received more asylum seekers in 2019 (UNHCR, 2020a). Besides that, Brazil is also the country with the largest economy, territory, and population in Latin America. I conducted 29 phenomenological interviews with refugees living in the states of São Paulo and Rio de Janeiro between March 27, 2020, and April 6, 2020, to understand how the outbreak of this pandemic affected them. Unlike other studies on asylum and the COVID-19 pandemic outbreak in which experts reflected on the effects of the pandemic on the refugee population (e.g., Jauhiainen, 2020; Júnior et al., 2020; Kabir et al., 2020; Kluge et al., 2020; Orcutt et al., 2020; Shammi et al., 2020; Vince, 2020), this article presents the reflection of refugees during the first 2 weeks of the epidemic in Brazil, when governors in these states adopted measures of quarantine, social distancing, and the closing of nonessential businesses and schools.

The interviews captured the perceptions of refugees during the beginning of the pandemic. This study offers a unique insight into the uncertainties people faced at that time considering their intersectional vulnerabilities. These findings contribute to understanding how refugees in the Global South living in urban contexts were affected by the outbreak of the biggest pandemic of this century. This article concludes that these refugees can be classified into four groups according to their labor vulnerability status in Brazil: (a) vulnerable refugees (unemployed and informal workers), (b) freelancer refugees (like designers and artists), (c) self-employed, and refugees with small businesses, and (d) employed refugees. These four categories, which appeared in the interviews, are differently affected by the pandemic based on the intersectionality of vulnerabilities. However, they face the same challenges as Brazilians in the same work/vulnerability situation. These categories dialogue with the findings of the survey of UNHCR Brazil and Cátedra Sérgio Vieira de Mello (CSVM; 2019) on the profile of the refugee population in Brazil considering employment. Nevertheless, the participants faced other problems connected to their situation as nonnationals living in Brazil. The pandemic aggravated challenges faced by refugees before the pandemic (e.g., lack of social networks and difficulties accessing rights and services) and created new challenges to this population (e.g., closure of borders and migration services).

## Asylum, Migration, and COVID-19

Even before the COVID-19 pandemic outbreak, migrant and refugee populations were already more vulnerable than nationals because they had higher chances of living in poverty, having mental health and chronic diseases (Truman et al., 2009), facing barriers to access the health system (Kluge et al., 2020; Truman et al., 2009) and information (Júnior et al., 2020; Kluge et al., 2020). Ventura (2015) explains that the vulnerability of refugees and immigrants also depends on individual characteristics such as age, gender, special needs, educational levels, and sexual orientation as well as structural causes like living and labor conditions, level of juridical protection, migration status, cultural and linguistic barriers, level of protection and risk of living in camps and detention centers.

Different experts pointed out the challenging conditions in refugee camps (Kluge et al., 2020; Shammi et al., 2020; Vince, 2020) and detention centers (Hargreaves et al., 2020; Keller & Wagner, 2020) that prevented people from adopting the WHO recommended measures necessary to fight COVID-19. These centers and camps are overcrowded with poor sanitation and health conditions (Jauhiainen, 2020), where refugees and immigrants lacked adequate health care services even before the pandemic outbreak. Additionally, refugees and migrants tend to be stigmatized (as risks or threats) and unjustifiably blamed for spreading viruses, which increases xenophobia, racism, and discrimination (Kluge et al., 2020; Ventura, 2015).

Politicians are using the pandemic to close borders and adopt racialized and discriminatory responses that exclude migrants and refugees (Devakumar et al., 2020). This bureaucratic discretion and the use of the pandemic to control migration flows lead to further marginalization of refugees (Sandvik & Garnier, 2020). The closure of borders and the adoption of mobility restrictions affected the rights of immigrants to enter and apply for asylum in many countries (Chiaretti et al., 2020; Riggirozzi et al., 2020). States also suspended national migration services (including refugee status determination procedures; Kluge et al., 2020), emissions of visas (including family reunification visas; Chishti & Pierce, 2020), rescue operations in the Mediterranean Sea and resettlement programs (Kluge et al., 2020).

Refugees, especially in urban areas, fear secondary consequences of the pandemic, such as restricted access to food, medicine, and essential services as well as the ensuing economic crisis (Betts et al., 2020). Refugees often work in areas with a high risk of losing their job. The International Federation of the Red Cross surveyed refugees in Turkey. Seventy percent of respondents lost their jobs when the pandemic started and 80% experienced an increase in daily expenses. Therefore, more than 50% of households had to adopt other mechanisms, such as borrowing money to cover expenses. The Movement of the Red Cross and Red Crescent identified this situation in other places like South and Central America (International Federation of the Red Cross, 2020). Many families left in the countries of origin depend on the money that immigrants and refugees send them to survive (Riggirozzi et al., 2020). The pandemic affected refugees and migrants’ capacity to send money abroad. IOM (2020) projects a decrease of nearly 20% in the flow of remittances compared with the past year, especially in Latin America due to the COVID-19 pandemic outbreak.

Another problem is the risk of funding for international and civil society organizations providing services for refugees in the context of economic and sanitary crises where more people require assistance and states have to invest more in their national health systems (Kabir et al., 2020). Civil society organizations that traditionally provide services to this population are prevented from providing those (Kluge et al., 2020) or have to adapt these services to online environments (Nisanci et al., 2020).

Studies have shown the importance of refugee-led organizations in the context of COVID-19 (Alio et al., 2020; Betts et al., 2020). Although refugee-led organizations are underfunded and do not receive the support of international donors, they have community-level trust, social networks, and adaptability that are necessary to support the refugee communities (Betts et al., 2020). They support refugees by providing information on COVID-19, acting as community health workers, tracking and monitoring the disease, supplementing capacity gaps (e.g., providing goods and services), and influencing social norms (Betts et al., 2020).

Researchers addressed the importance of including migrants and refugees in global responses to the COVID-19 pandemic, protecting their human rights (including the right to health), granting them access to health care without discrimination, and assuring that they will not face sanctions of state authorities (Brandenberger et al., 2020; Kluge et al., 2020; Orcutt et al., 2020). Studies about other health emergencies highlighted the need for transparent migrant and refugee-inclusive information (Truman et al., 2009) and the importance of engaging and adequately involving migrant and refugees in prevention, preparedness, and responses to combat pandemics of HIV/AIDS (Spiegel & Nankoe, 2004) and influenza (Truman et al., 2009). Hence, not protecting refugees and migrants may create negative externalities for the society.

### The Pre-COVID-19 Situation of Refugees in Brazil

Brazil is the largest and richest country in Latin America. Brazil recognized near 38,000 Venezuelans as refugees in 2019,^2^ considering the situation of severe and generalized violation of human rights in the country (Delfim, 2020). Most Venezuelans arrived in the state of Roraima where the Brazilian federal government coordinated Operation Welcome (*Operação Acolhida*) in partnership with international and civil society organizations (UNHCR, 2020b). Currently, Brazil has 43,000 recognized refugees from more than 80 nationalities (Delfim, 2020), including Syria, the Democratic Republic of Congo, Colombia, Palestine, Pakistan, Mali, Iraq, Angola, Afghanistan, and others (*Comitê Nacional para os Refugiados* [CONARE], 2019). Before the recognition of Venezuelans as refugees, São Paulo and Rio de Janeiro, respectively, were the states where most refugees used to live (UNHCR & CSVM, 2019).

The UNHCR recognizes the Brazilian asylum law as progressive (Jatobá & Martuscelli, 2018). The National Committee for Refugees (CONARE), a tripartite organization comprising representatives of the Brazilian government, civil society organizations and the UNHCR (with no vote), is responsible for analyzing the asylum claims and defining public policies for refugees and asylum-seekers (Jatobá & Martuscelli, 2018). Nondiscrimination is a principle in the Brazilian Federal Constitution (1988), and refugees have the same rights as Brazilians according to the Asylum Law (Law 9474/1997) and the Migration Law (Law 13445/2017). Refugees and asylum seekers have access to the Brazilian public health system (*Sistema Único de Saúde*), education, and social assistance programs. They receive the Brazilian identification document for immigrants—*Registro Nacional Migratório*—and the work permit (*Carteira de trabalho*).

However, refugees and asylum seekers face challenges to accessing their rights in Brazil. Brazil has no federal integration policy, no national program to teach Portuguese or culturally and linguistic adapted services to this population (Moreira, 2014). These populations also face challenges to access bank services and some public systems that ask for the number of the Brazilian identification number (*Registro Geral*) that only Brazilians have (Moreira, 2014).

Refugees also struggle to access the labor market. In 2018, UNHCR Brazil and Cátedra Sérgio Vieira de Mello (CSVM) conducted a representative survey with 487 refugees living in Brazil (UNHCR & CSVM, 2019). They showed that refugees in Brazil were, on average, higher educated than the Brazilian population. However, they faced barriers to have their diplomas recognized and to enter the Brazilian labor market in positions according to their skills, having a higher percentage of unemployment than nationals. That is, 68.2% of them were not using their professional skills at the time of the survey, 19.5% were looking for a job, and 25.2% were out of the labor market, while 17.9% were self-employed (UNHCR & CSVM, 2019).

According to data from UNHCR Brazil (UNHCR, 2020b), only 10% of the Venezuelans in Brazil were in the formal market (while 34% of Brazilians were in the formal market) before the pandemic outbreak. Employed Venezuelans worked in services “such as restaurants, coffee shops, and snack bars, in addition to retail trade as well as some industrial and agroindustry sectors such as construction and meatpacking” (UNHCR, 2020b, p. 2). These are areas which are significantly affected by the economic crisis due to the outbreak of the COVID-19 pandemic.

Friedrich and Jasper (2020) reflect on the different vulnerabilities of migrants^3^ considering characteristics previously the COVID-19 pandemic, like employment status and barriers to access rights like language and disinformation. Another critical barrier to access services pre-COVID-19 was discrimination. Indeed, 41% of the refugees surveyed by UNHCR and CSVM (2019) mentioned that they felt discriminated against: First, because they were foreigners, and second, because they were Black. African refugees had a higher chance of feeling discriminated against since they are Black and foreigners. Previous studies have documented African refugees facing discrimination in accessing the Brazilian public health system (Horta et al., 2019) and local attempts to limit their access to these services to Venezuelans in the city of Boa Vista (Riggirozzi et al., 2020). Other studies showed the lack of specific training to public servants about the situation of refugees and general lack of culturally and linguistic specific public policies to this population (França et al., 2019).

Brazil did not adopt effective measures to control the pandemic. In January 2021, the country was among the most affected by COVID-19 with more than eight million infected people and 207,000 deaths (WHO, 2021). The Brazilian President Jair Bolsonaro was internationally criticized due to his lack of responses (Ponce, 2020). The federal government closed the borders to foreigners and asylum-seekers (especially Venezuelans). Migration deadlines, CONARE meetings, and services at the Federal Police were temporally suspended (Jubilut et al., 2020). The federal government did not adopt the measures recommended by WHO. In fact, Bolsonaro minimized the pandemic, was against measures of social distancing, closure of the commerce and quarantine, urged the population to use medicines that were not scientifically approved to fight COVID-19, and undermined the efforts of state governors (Jubilut et al., 2020). The federal government created a temporary emergency cash benefit to support people in more vulnerable economic situations called *Benefício de Assistência Emergencial*. São Paulo and Rio de Janeiro were two states that closed nonessential services and adopted WHO measures of social distancing in the beginning of the pandemic in Brazil. While these measures helped to control the infections in these states, the population was receiving mixed signals since the federal government and the state governments had different approaches to fight this virus (Jubilut et al., 2020).

## Method

This study follows the phenomenological perspective. Initially proposed by Edmund Husserl (1913/1962) and further developed by several authors across different disciplines,^4^ this theoretical approach studies the phenomena of the reality accessing the experiences of those who live them. That is, understanding a phenomenon means considering the living experience of people. This process involves the idea of bracketing, or the reflexive process of the researcher to understand their ideas, preconceptions, and judgments and try to leave them outside to access the phenomenon as it is/was lived by people (Husserl, 1913/1962). This process of bracketing allowed me to understand the different power relations between the researcher (White, female, Brazilian) and refugees (non-White and nonnationals). I adopted intersectionality as an analytical approach as recommended by Kassam et al. (2020). I understand intersectionality “as a worldview that aims at disrupting inequity and revealing processes of power, privilege, and disadvantage among complex populations” (Kassam et al., 2020, p. 4). Intersectionality involves the “confluence of multiple identities in each individual” that occupy multiple social identities and social categories (like gender, class, sexual orientation, nationality). Each one of these categories puts this person in an “oppressed or a privileged position” (Vervliet et al., 2014, p. 2025).

The intersectionality approach helps to understand how the refugees lived the COVID-19 pandemic outbreak by considering how the many identities that they have (class, race, and nationality) put them in a more vulnerable position. Refugees are a complex population because they have different layers of vulnerability that make their rights more easily violated: nonnationals (without vote), forced displaced populations, numeric minority, non-Portuguese native speakers, and perceived as different because of their race, nationality, religion and cultural traditions. While other studies reflected on intersectionality and refugees (e.g., Hayes, 2018; Lee & Brotman, 2013; Vervliet et al., 2014; Yacob-Haliso, 2016), this research contributes to understand how being a refugee (with all the multiple identities involved) facing the outbreak of the COVID-19 pandemic in Brazil involves additional challenges that are not faced by the Brazilian population.

Qualitative studies during this pandemic are useful to “underscore the complexities that vulnerable people face during epidemics and [ . . . ] to capture that complexity to understand the scope and impact of an outbreak” (Teti et al., 2020, p. 1). The studies on asylum and the COVID-19 pandemic show how experts and practitioners understand the impact of the COVID-19 pandemic outbreak on refugees. However, there is a lack of studies which grant space for refugees to reflect on their experiences and issues that matter to them during the pandemic. There is a need to understand if the reflections of experts in the area represent the challenges that refugees actually face living the pandemic in a Global South country in Latin America like Brazil.

Refugees are experts of their experiences (Hynes, 2003). Alio et al. (2020, p. 373) explains that, “research on the impact of COVID-19 on refugees must include refugees [ . . . ]. The inclusion of refugees will lead to research that is better informed by the realities it seeks to explain and more likely to alleviate the suffering it studies.” Hence, it is necessary to listen to their narratives and voices if we aim to understand how they live the biggest pandemic of this century. Therefore, I employed the methodology of “phenomenological interview” (Høffding & Martiny, 2016) to answer the question: How are refugees affected by the COVID-19 pandemic outbreak in Brazil? Based on the literature, refugees (due to their multiple intersectional identities) will be differently affected by the COVID-19 pandemic outbreak because they are nonnationals who performed a forced migration. Phenomenological interviews understand and value the expertise and the lived experiences of their participants (Høffding & Martiny, 2016). That is, people who are experiencing the pandemic as refugees in Brazil can better understand this phenomenon. The semistructured interviews allowed me to capture their reflections, thoughts, and lived experiences while considering them as conscient agents.

I conducted 29 semistructured phenomenological interviews with forcibly displaced people with refugee status in Brazil between 27 March, 2020, and 6 April, 2020. Since refugees are a hidden and hard to reach population in Brazil, I used snowball sampling to recruit the participants (Noy, 2008). Refugee leaders gave me the contact of other informants and I conducted interviews until the saturation point where no new information was being provided. The Interview Guide had questions involving Information on the refugee, Information on COVID-19, Work, Contact with family and friends, Answers from the Brazilian authorities, and Advices and Future thoughts (See Online Appendix). Questions like “How are you dealing with the COVID-19 pandemic?” “How are you feeling at this moment?” and “How do you think that this pandemic affects your life?” allowed the refugees to reflect and express how they were living in the first 2 weeks of the pandemic. All in all, it allowed me to assess which areas were sensitive for refugees in Brazil and why. All interviews were conducted by phone through the WhatsApp app to respect social distancing measures due to the COVID-19 pandemic.^5^ On average, the interviews lasted 38 minutes and 27 seconds, with a total of 18 hours, 35 minutes, and 4 seconds of recorded material. While interviews conducted by phone tend to be shorter than face-to-face interviews, they also allow researchers to assess people’s lived experiences (Irvine, 2011).

The refugees were mostly male, young, and living in the states of São Paulo and Rio de Janeiro. They came from the Democratic Republic of Congo, Syria, Venezuela, Mali, Cameroon, Guinea, and Guyana. Table 1 in the online appendix shows the participants’ characteristics and the length of the interviews. The documents of this study including the Recruitment Text, the Study Background, and the Interview Guide with the oral informed consent script are also available in the online appendix.

The research followed the ethical recommendations of the Code of Ethics of the International Association for the Study of Forced Migration (IASFM, 2019). This document reinforces the importance of “do no harm” research, respecting the confidentiality and privacy of the participants and guaranteeing that their participation is voluntary and based on informed consent. I also followed the principles of autonomy, equity, diversity, competence, and partnership as recommended by IASFM (2019). All refugees gave their informed oral consent to be part of the research and to have the interview recorded. All interviews were conducted (without interpreters since I speak Portuguese, English, and French), transcribed, and coded by me using Atlas.ti8. These two measures avoided privacy and confidentiality breaches. I coded these interviews with descriptive coding (“Assigns labels to data to summarize in a word or short phrase—most often as a noun—the basic topic of a passage of qualitative data”; Saldaña, 2009, p. 261), emotion coding (“Labels the emotions recalled and/or experienced by the participant, or inferred by the researcher about the participant”; Saldaña, 2009, p. 263), and versus coding (“Identifies in dichotomous or binary terms the individuals, groups, social systems, organizations, phenomena, processes, concepts, etc., in direct conflict with each other”; Saldaña, 2009, p. 268). After that, I produced thematic coding memos. The results present the themes that emerged from the analysis focusing on three different types of challenges that refugees faced because of the COVID-19 pandemic in Brazil due to their intersectional multiple identities.

## Results

The Results sections show how the refugees interviewed were affected by the pandemic according to their intersectional identities and took into consideration situations that affected refugees and Brazilian (like labor and economy) considering their class identity; situations that refugees faced before the pandemic aggravated due to COVID-19 pandemic outbreak and new situations that refugees started to face because of the pandemic involving their identity of forcibly displaced nonnationals.

### Challenges Faced by Refugees and Brazilians During the Pandemic in Brazil

The economic consequences of the COVID-19 pandemic outbreak affected refugees and Brazilians in the same way according to their labor vulnerability classification. That is, this first part of the results considers the class identity where access to labor and social protection puts refugees and Brazilian in a more oppressed or privileged situation to face this pandemic. This classification is useful to understand how refugees are affected by the pandemic considering their social identity based on work. The refugees that I interviewed could be classified into four categories considering their current work situation in Brazil. These four categories dialogue with the results of the survey of UNHCR and CSVM (2019). Each of these categories faces specific challenges to deal with the pandemic. Table 1 presents illustrative quotes from refugees in each one of these categories.

**Table 1.** Four Different Categories of Refugees Based on Labor Vulnerability.

Category of refugee	Illustrative quote
1. Vulnerable refugees	I am unemployed, I already got a job, but with this coronavirus, I am not able to work. I have done an interview. I already delivered the documents to start [the job]. It was going to start last week, but last week the quarantine started here. (Congolese refugee, 28 years, Interview, March 31, 2020).
2. Employed refugees	The situation is very complicated, you know, like, my uncle worked [on a company], and on the last day they fired him because the company had no way to pay him, you know? But. . . I am still working, but I do not know what the future is going to be like, right? Because we are at home. (Congolese refugee, 20 years, Interview, April 4, 2020).
3. Freelancer refugees	It is not a contract job; it is more like freelancing. I do not have much of the rights. But it was good for me for now. But yeah, with the coronavirus, everything has stopped, even this job was canceled. So at least what they were paying is enough for the next two months. The job is frozen now. (Syrian refugee, 30 years, Interview, March 31, 2020).
4. Self-employed refugees	The coronavirus zeroed everything we did during these five years in Brazil. Everything we planted, we lost it all due to coronavirus because we do not have a restaurant. We only work with events and orders. So no income is entering now. (Syrian refugee, 40 years, Interview, March 27, 2020).

*Note.* Elaborated by the author based on the 29 semistructured phenomenological interviews with refugees living in the states of São Paulo and Rio de Janeiro between 27 March, 2020, and 6 April, 2020.

The first category involves *vulnerable refugees*: They are unemployed people and/or illiterate and/or are typically elderly. Many of them live in shelters, *ocupações* (occupations, abandoned places where families live), and *favelas* (slums). Like Brazilians living in vulnerable situations, the pandemic prevented them from looking for a job and from working since many of them worked in the informal market with no social protection. Many of them were dependent on donations of food and other items and they feared not having money to pay their bills.

The second category is *employed refugees*. They are part of the formal labor market and have access to social protection connected to work. Like employed Brazilians, many of them were doing home-office (like language teachers teaching online), others were at home waiting for a decision from their companies, and many were essential workers in supermarkets, logistics, shelters, and food businesses. They had social protection in Brazil but they were worried about the future of the Brazilian economy and about losing their job in the ensuing economic crisis caused by the pandemic.

The third category is *freelancer refugees*. They are younger, have skills and higher education. Most of them were liberal professionals (freelance professionals) like designers, artists, artisans. Like Brazilians in this area, some were able to continue working from home. However, many had to stop working. If they had some savings, they were in a good situation and could resist the closure of many businesses. However, they were worried about the length of the pandemic. Many had no savings and were worried about how to pay the bills.

The fourth category is *self-employed refugees*: They have small businesses (most in the area of food services). Like Brazilians in the same position, they were severely affected by the pandemic. Many events, fairs, and parties they usually catered to were canceled. Although many were delivering food, they were worried about the duration of the pandemic because sales were slow, and they needed to pay their bills. Many of them produced food from their houses, but some had small restaurants, and they had to continue paying these bills.

The pandemic also brought other challenges to Brazilians and refugees. Housing is a central issue in Brazil. Rent represents a considerable part of the income of many families in the country. A significant part of the Brazilian population lives in slums, or collective units lacking hygiene measures and with no possibility of social distancing. In a survey conducted with 487 refugees, 26.2% (*N* = 129) of refugees lived in collective units, and 90.6% (*N* = 441) lived in rented houses (UNHCR & CSVM, 2019). Another problem that refugees and Brazilians faced due to their social identities connected to class was not being able to pay for their bills. However, 67% of the refugees (*N* = 326) said their income level was not enough to cover their expenses even before the COVID-19 pandemic outbreak (UNHCR & CSVM, 2019). Some of the interviewees were worried since they were not working and could not pay their rent and bills:

I am in charge of the family, so sometimes I do not sleep at night, can you believe me? I am thinking about how I am going to pay the rent, how I am going to solve these things. [ . . . ] They want the rent, but I cannot afford it. (Syrian refugee, 40 years-old, self-employed, Interview, March 27, 2020)

Hygiene products like alcohol and masks added an extra burden in some refugee families’ budgets. In some cases, refugees and Brazilians lost income due to the pandemic which put them in hard situations of having to choose to buy hygiene supplies or food. That was the case of this unemployed 25 years old Congolese refugee:

if you have a family that has ten reais,^6^ and you must decide to have breakfast or buy alcohol [for hygiene measures], what would you rather buy? (Interview, April 1, 2020)

A third situation that has affected refugee and Brazilian families (especially women) is the closing of schools. Children are staying at home and women are expected to take care of them full time. Children were having more meals at home that they used to have in Brazilian public schools which affected the food needs of low-income families. That was the case of this unemployed 39 years-old Congolese woman: “children are eating every day. [ . . . ] You must receive food support for children who study. I do not know what I am going to do. . . . When schools are opening, I do not know” (Interview, April 4, 2020). Despite that, during these interviews, many refugees reflected on the situation of people that were more vulnerable than them like people living in the streets, families in *ocupações*, and recently arrived asylum-seekers.

### Challenges of Refugees Aggravated by the Pandemic

Refugees face different challenges due to their social identity as nonnationals even before the COVID-19 pandemic outbreak. During the interviews, the refugees concluded that many difficulties faced by them were aggravated due to the pandemic, especially the access of services and rights and the fact that they do not have much information, social networks, and resources. One self-employed 33-year-old Syrian refugee argued that “we do not have many things; [ . . . ] There are many things that we do not know. I think that for refugees, it [facing the pandemic] is more difficult than for Brazilians because everything is new for us” (Interview, March 30, 2020).

Refugees lack social networks to support them in moments of crisis. During one of the biggest pandemics of the century, this lack of support was aggravated. A 40-year-old Congolese refugee living in Brazil since 2013 explained that:

most immigrants are here without a root. Because plants need roots in the land to grow. Brazilians have roots here because they have an uncle, brother-in-law, nephew, there is always someone they can ask [for help]. However, an immigrant, he is unusual; he is the tree that was placed without a root. (Interview, April 3, 2020)

Although some refugees said they had Brazilian and foreign friends (some say they were not friends, they were more like colleagues), they felt that they did not have somebody they could count on. The pandemic highlighted the importance of social networks and the fact that refugees had no people to ask for help:

you can say “So my mom, my grandma, my dad has a house I can live there.” Then you go there, and you have some relatives there who have a house. But imagine us? Living here in this country that we do not have even an uncle. You are two, three months without paying rent. Who will let you live without paying [the rent]? (Congolese refugee, 20 years, living in Brazil since 2012, Interview, April 4, 2020)

Refugees were already facing challenges to access their rights to social assistance and health care before the pandemic. During the pandemic, refugees were uncertain if they could access the emergency benefits the government was discussing:

the government is discussing some help. [ . . . ] Several immigrants and refugees are in this [uncertain] situation. How will we benefit from this aid? [ . . . ] I have no guidance; I am looking to do this registration to get this benefit. There are several refugees in this situation. [ . . . ] Maybe we will not be able to fit into this help package that the government is discussing. (Mali refugee, 39 years, Interview, March 30, 2020)

Lack of access to culturally and linguistically adapted information prevents refugees from accessing rights and services. This situation was aggravated with the pandemic, when information was necessary to keep people healthy and safe. There was no information on COVID-19 provided to refugees in a way that was useful to them: “I do not see any specific information that is being passed on to refugees. No message, [ . . . ], we did not receive anything” (Congolese refugee, 30 years, Interview, March 30, 2020). “I do not see anything for refugees from organizations or even the media. I did not see anything” (Syrian Refugee, 30 years, Interview, March 31, 2020).

Refugees also faced difficulties accessing information and benefits due to linguistic barriers: “We want to access information; information is something that helps many refugees. So, the correct information helps a lot. [ . . . ] Many people do not understand the Portuguese language; all of this is a little complicated, right?” (Mali Refugee, 38 years, Interview, March 30, 2020). “There is also the issue of language; there are many [refugees] who do not speak Portuguese, there are many who suffer from xenophobia or racism” (Syrian Refugee, 27 years, Interview, April 3, 2020). Refugees reflected that this lack of information was aggravated due to the pandemic. It was dangerous because it increased their risk of believing in fake news, falling in schemes to steal their data (promising to register in the governmental emergency benefit), and suffering more uncertainty about the disease and the future.

Discrimination and xenophobia when accessing the public health care system were other challenges aggravated in the context of the COVID-19 pandemic. An employed 23-year-old Congolese refugee feared being discriminated against if he got sick: “we pray not to have this disease because, in our mind, we know that it will be difficult for us to be treated like the Brazilian people” (Interview April 1, 2020). Another 28-year-old Congolese refugee feared going to the hospital because he faced discrimination when he arrived in Brazil in 2014:

I got a cold, but it is not COVID-19. But I cannot go to the health center, because something happened in 2014. When I got here, I had the flu. [The doctors] did not tell me anything, they said I had Ebola, you know? (Interview, March 31, 2020)

Branco (2020) showed examples of immigrants and refugees who faced prejudice and racism when trying to access the Brazilian health system during the pandemic. The author also discussed that there were few statistics on refugees, immigrants, and COVID-19 in Brazil which invisibilized these populations and prevented decision makers from understanding their needs and infection and mortality rates.

### Challenges of Refugees Created by the Pandemic

Although the interviewees have been living in Brazil on average for the past 6 years and 9 months (ranging from 2 years and 4 months to 12 years), the pandemic has created new challenges for this population due to their social identity of forced displaced nonnationals and nonnative Portuguese speakers. Many refugees perceived that recently arrived people and people who were still waiting for their asylum procedures were in a more vulnerable situation than them. However, the pandemic created specific problems for refugees who are nonnationals and have families living in their countries of origin.

Migration services were suspended in Brazil due to the pandemic: naturalization appointments, asylum interviews, CONARE meetings, family reunification procedures, and visas emissions. Many refugees had their appointments in the federal police (responsible for many migration services) to do their naturalization procedures, or their documentation canceled. Although Brazil extended the migration deadlines, this created much uncertainty, mainly because refugees had to wait a long time for their appointments. One 37-year-old Venezuelan refugee reflected that “the process in the Federal Police is very complicated. [Due to] scheduling issues and lack of information [ . . . ] And because of this pandemic, our appointment was canceled, and we are in the limbo.” (Interview, April 6, 2020).

The pandemic also created longer separation times and uncertainty for people waiting for family reunification for their relatives that lived abroad. It was not clear if refugee family members (who already had the visa) would be able to enter in Brazil because the Brazilian borders were closed for nonnationals. A 40-year-old Congolese refugee explained:

You must not forget that there are also immigrants whose families were away. They were still collecting money, and when they got the money to buy the ticket, Brazil closed the door. Can that person board that plane too? Or not? [ . . . ]. Are these people being identified? Does Brazil know their situation? (Interview, April 3, 2020)

He also reflected that refugee families abroad depend on money from refugees living in Brazil to survive: “When someone in Brazil does not send money, [the family] gets hungry. They live outside Brazil but ‘eat Brazilian money’”^7^ (Interview, April 3, 2020). The UNHCR and CSVM (2019) showed that 49.9% of refugees in Brazil sent money abroad. Another problem caused by the pandemic was that agencies that send money abroad and agencies that do international phone calls were closed. They were not considered essential services (e.g., supermarkets or pharmacies). This was harming refugees’ capacity to communicate with their families abroad and to send them money:

Did they think that these agencies have to stay open when they said they were going to close everything? So do you think that a person will be at peace at home, sleeping, eating, knowing that she has not paid the rent [of their left behind family]? [If] the daughter gets sick, she must send money, but there is no way to send it. (Congolese Refugee, 40 years, Interview, April 3, 2020)

Another 25-year-old Congolese refugee had no communication with his mom and reflected how it was hard for him that the international phone places were closed due to the pandemic: “Now you cannot leave, it is very difficult. I could even go to the gallery to ask someone for help to call my mom, right? Now, it is closed” (Interview, April 1, 2020).

Another problem created by the pandemic was that organizations that helped refugees and asylum-seekers were also closed. Although they were trying to provide services remotely, refugees lost their point of reference, since these organizations were not “working”:

Today, everything is not working; everything is not working. And there are recently arrived people here; maybe some are not formalized, how will they make a living, right? Almost everything stopped. Worse, there is no assistance now. Do you understand? There is no assistance, no work, so. . . . This is complicated for these people. (Congolese Refugee, 30 years, Interview, March 30, 2020)

Finally, refugees lived the pandemic twice: they experienced the effects of the COVID-19 pandemic outbreak (including the ineffective governmental responses) in Brazil where they had to worry about their health and wellbeing (and the relatives that were in Brazil). But, at the same time, they worried about their families living through the pandemic in their countries of origin. All interviewees had family members living abroad and were worried about their health and wellbeing. The COVID-19 pandemic affected countries in different times. Some states had more resources and/or were better able to address the pandemic in an effective way. Most interviewees came from Syria, the Democratic Republic of Congo, Mali, and Venezuela. These countries have been facing armed conflicts, humanitarian emergencies, and human rights violations. These countries were facing an even worse economic and humanitarian situation than Brazil. The respondents were worried about the situation of their families since their origin countries lacked resources to fight the pandemic:

My concern is my family. I am here worried about my life. I am also worried about my family because there is no structure in Mali. The country has no structure to face this disease. It does not have. It is a country that has less than fifteen respirators [for] the whole country. (Interview, 39 years, March 30, 2020)

There are no tests and everything. . . after all, the country has been at war for nine years. So, there is no structure to face it, that is a fact. There is no way to control the coronavirus in Lebanon, Iraq, or Syria, so forget it. (Syrian Refugee, 27 years, Interview, April 3, 2020)

The fact that they could not communicate properly with their families due to technical limitations (e.g., lack of internet access in the countries of origin) and that they could not send money abroad exacerbated their worries about their families:

it is difficult, very difficult . . . I do not even know how my parents are doing. . . . If things do not get better, then I will only hear one day that my parents no longer exist. . . . Because it is very difficult! I am almost two months, three months without knowing anything about them. (Congolese refugee, 25 years, Interview, April 1, 2020)

## Discussion

As many different complex populations in Brazil (e.g., Black Brazilian, *quilombolas*,^8^ indigenous), refugees in Brazil perceived that they were abandoned or neglected in the responses to the COVID-19 pandemic which increased their feeling of uncertainty, hopelessness, and fear. They perceived that the Brazilian government did not consider refugees (and their intersectional multiple identities) in the responses to the pandemic: “I never heard a person in Brazil speaking exclusively about refugees and immigrants. We are alone. We do not have any special coverage; we have not had anything special until now” (Mali Refugee, 39 years, Interview, March 30, 2020). “And from what I watched the interviews of the most powerful people in the country, nobody is talking about the refugees.” (Congolese refugee, 28 years, Interview, March 27, 2020). An intersectionality approach allows us to understand how the outbreak of the COVID-19 pandemic affects refugees considering their multiple social identities. This research contributes to the literature on intersectionality and asylum by showing how different refugees’ social identities put them in a situation of oppression considering their class and access to work and their identity as forced displaced nonnationals and non-Portuguese native speakers. This makes refugees a population that face three types of challenges: (a) same challenges as Brazilians due to their labor vulnerability social identity, (b) challenges aggravated by the pandemic due to their social identity of nonnationals including access to information and services like health care and social assistance, and (c) new challenges due to their social identity of forced displaced nonnationals including closing of migration services, difficult to communicate and reunite with their families and the feeling of living the pandemic twice.

As a practical consequence, refugees were a left-behind group during the first 2 weeks of the pandemic in Brazil. While the literature on the COVID-19 pandemic and refugees had already reflected challenges that the pandemic could bring to refugees, the interviews showed that the multiple social identities of refugees were neglected in the Brazilian responses to COVID-19, which created different types of challenges connected to the pandemic. The results sections highlighted that refugees face the pandemic the same way of Brazilians considering their labor vulnerability classification. Vulnerable refugees, freelancer refugees, employed refugees and self-employed refugees faced the consequences of the pandemic differently considering their access to stable income and social protection. Many refugees and Brazilians that occupy this same social identity were affected by the decrease of their income and impossibility to work due to the pandemic. This situation affected their capacity to pay their bills and rent (since housing is a main issue in Brazil). The closure of schools has also affected all people in Brazil, especially low-income families. This topic appeared more in the narratives of female refugees, indicating the gender expectations that women were supposed to take care of children.

At the same time, the pandemic has aggravated challenges that refugees already face in Brazil due to their social identity of nonnationals. Refugees lacked social networks that could help them during this difficult time of crisis. Refugees had a hard time to access information on COVID-19 that was linguistically and culturally adapted to them. Moreover, they feared discrimination and xenophobia when accessing the health care system. Countries should consider the different challenges refugees face during the pandemic due to their intersectional identities and how the lack of access to information and health care contributed to this health emergency. While previous studies reflected on difficulties to access information and services, the importance of social networks to give support during times of crisis lacked in previous reflections on migration, asylum, and COVID-19.

Finally, the pandemic created new challenges for refugees living in Brazil independently of how long they have been living in the country due to their multiple identities that put them in an oppressed situation. The closure of borders, migration services, organizations providing services to this population, agencies that send money abroad, and international phone companies seriously affected the lives of refugees. It was the first time that the participants faced this situation in Brazil. The interviews showed that these services were essential to refugees. In that sense, the government should have considered how measures of closure of borders could affect this population, in particular people who were in the middle of their family reunification procedures. Since these measures did not consider the specificities of refugees, the pandemic created new problems that increased their feelings of uncertainty, fear, and hopelessness. Finally, refugees come from countries with armed conflicts, persecutions, and humanitarian emergencies. These places are less equipped to face the biggest pandemic of the century than Brazil. The pandemic created this feeling that refugees were living the pandemic twice since they had to worry about their health and wellbeing in Brazil and about their relatives living in the origin countries. The importance of essential services to the refugee population and this perception of “living the pandemic twice” did not appear in the previous reflections on refugees and COVID-19.

The interviews indicated the importance of understanding how the pandemic affected refugees considering their multiple intersectional identities which involve the same challenges faced by Brazilians, challenges aggravated by the pandemic and new challenges created by the pandemic. Listening to the refugees themselves showed that many reflections on how the COVID-19 pandemic affected refugees’ dialogue with their priorities during this crisis. However, new challenges and worries appeared in the phenomenological material. In that sense, the empirical material may help decision-makers and practitioners to understand how the pandemic affected this population considering their multiple different social identities that put them in an underprivileged situation making sure that their specific needs and worries (connected to each one of their social identities) will be considered in the responses to the pandemic in Brazil.

## Conclusion

Refugees living in the states of São Paulo and Rio de Janeiro reflected on their own experiences as forcibly displaced people living through the first 2 weeks of the COVID-19 pandemic outbreak in Brazil. The phenomenological interviews showed that refugees, in some aspects, are equally affected by the COVID-19 pandemic as Brazilians considering their different labor vulnerability social identity. However, the international forced displacement negatively affects refugees while facing the pandemic in Brazil. This study presented the impacts of the COVID-19 pandemic outbreak on refugees living in urban areas in a Global South country considering their intersectional identities. Refugees faced challenges before the pandemic that were aggravated by it and challenges that were created by the responses to this new disease that did not consider their multiple social identities. Before COVID-19, refugees already lacked social networks and faced difficulties to access rights and services (including benefits and health care). Therefore, the pandemic aggravated this area. Furthermore, the responses to the pandemic created additional problems that refugees did not have in Brazil like the closure of borders and “essential” services and the perception that refugees were “living the pandemics twice” since their countries of origin lacked resources to fight the pandemic.

This study reinforces the need to understand how forcibly displaced people in the Global South are affected by pandemics and responses to them adopting an intersectionality approach, especially in situations that are not refugee camps, like Brazil. Granting refugees the possibility to reflect on their experiences provides valuable insight on how to involve and engage them in responses to global health emergencies. This article captures a unique photography of refugees facing the initial weeks of the pandemic in Brazil. Further studies should consider the perception of refugees after living months of the pandemic in Brazil. This reflection was a first empirical attempt to capture their lived experiences considering their multiple social identities. Other works should consider different identities of this population like gender and age for example. Another research agenda could consider the experience of refugees who had COVID-19 or had a relative that got this disease. Their perceptions navigating the Brazilian public health system during the pandemic can also add to the reflections on how refugees are affected by the COVID-19 pandemic in Brazil.

## Supplemental Material

sj-pdf-1-abs-10.1177_00027642211000402 – Supplemental material for How Are Forcibly Displaced People Affected by the COVID-19 Pandemic Outbreak? Evidence From Brazil Click here for additional data file. Supplemental material, sj-pdf-1-abs-10.1177_00027642211000402 for How Are Forcibly Displaced People Affected by the COVID-19 Pandemic Outbreak? Evidence From Brazil by Patrícia Nabuco Martuscelli in American Behavioral Scientist

## Notes

1. According to the WHO (2021), Brazil was the third most affected country by COVID-19 in the world in number of infections, after the United States of America and India (respectively) in January 2021.

2. For detailed data on refugees in Brazil see https://app.powerbi.com/view?r=eyJrIjoiNTQ4MTU0NGItYzNkMi00M2MwLWFhZWMtMDBiM2I1NWVjMTY5IiwidCI6ImU1YzM3OTgxLTY2NjQtNDEzNC04YTBjLTY1NDNkMmFmODBiZSIsImMiOjh9

3. All migrants in Brazil are protected according to the Brazilian Migration Law (13.445/2017). Refugees are forced migrants that were recognized as refugees according to the definition and procedure of the Brazilian Asylum Law (9474/1997). Hence, all refugees are migrants, but not all migrants are refugees.

4. For further discussions on phenomenology across different disciplines see, among others, Davis (1971), Laverty (2003), Giorgi and Giorgi (2003), Lopez and Willis (2004).

5. For a comprehensive discussion on how to conduct primary data collection during the pandemic see Samuels (2020).

6. The Brazilian Money is called Brazilian real. *Reais* is the plural of real in Portuguese.

7. “Eat Money” is a Brazilian expression that means to spend money. When the interviewee says that families “eat Brazilian money” this means that these families need the Brazilian money to survive (to eat) and the money they spend in their countries comes from Brazil.

8. *Quilombolas* are communities’ descendants of former slaved African people in Brazil.
