# Supplementary material for: How Are Forcibly Displaced People Affected by the COVID-19 Pandemic Outbreak? Evidence From Brazil
Source: Am Behav Sci. 2021 Sep;65(10):1342–64. doi: 10.1177/00027642211000402 (PMC7992097; doi:10.1177/00027642211000402)
Supplement: sj-pdf-1-abs-10.1177_00027642211000402 – Supplemental material for How Are Forcibly Displaced People Affected by the COVID-19 Pandemic Outbreak? Evidence From Brazil [file sj-pdf-1-abs-10.1177_00027642211000402.pdf]

Online Appendix –

How are forcibly displaced people affected by the COVID-19 pandemic outbreak?

Evidence from Brazil

Summary

Profile of the participants of the Study Refugees and COVID-19 in Brazil. 2

Table 1 - Profile of the participants 2

Documents of the Study 4

Recruitment text 4

Study Background 5

Interview Guide with oral informed consent script 6

Profile of the participants of the Study Refugees and COVID-19 in Brazil.

Table 1 - Profile of the participants

Sex	Age	Nationality	State	Risk group[1]	People living with the participant	Labor-vulnerability Category	Year of arrival in Brazil	Language of the interview	Date of the interview	Duration of the interview (minutes)
Male	48	Syria	São Paulo	No	Wife	Employed (essential service)	2015	Portuguese	27/03/2020	32:25
Male	40	Syria	São Paulo	No	Wife and children	Self-employed (food area)	2014	Portuguese	27/03/2020	25:37
Male	28	Democratic Republic of Congo	São Paulo	No	Wife	Employed	2015	Portuguese	27/03/2020	30:42
Male	35	Democratic Republic of Congo	São Paulo	No	Wife and children	Vulnerable (unemployed)	2014	Portuguese	28/03/2020	53:16
Male	47	Syria	São Paulo	No	Wife and children	Self-employed (food area)	2013	Portuguese	28/03/2020	27:41
Male	38	Mali	São Paulo	No	Friends	Employed	2012	Portuguese	30/03/2020	26:47
Male	39	Mali	São Paulo	No	Brother and wife	Vulnerable (unemployed)	2013	Portuguese	30/03/2020	31:38
Male	33	Syria	São Paulo	No	Wife and children	Self-employed (food area)	2014	Portuguese	30/03/2020	32:08
Male	30	Democratic Republic of Congo	São Paulo	No	Brother, sister-in-law, and nice	Employed (essential service)	2013	Portuguese	30/03/2020	34:47
Male	34	Democratic Republic of Congo	São Paulo	No	Alone	Employed[2] (essential service)	2014	Portuguese	30/03/2020	25:57
Male	34	Guinea	São Paulo	No	Alone	Employed	2013	Portuguese	30/03/2020	27:35
Male	33	Cameroon	São Paulo	Yes	Alone	Employed	2014	Portuguese	30/03/2020	27:01
Male	40	Democratic Republic of Congo	São Paulo	Yes	Wife and children	Employed	2013	Portuguese	31/03/2020	43:25
Male	28	Democratic Republic of Congo	São Paulo	No	Alone	Vulnerable (unemployed)	2014	Portuguese	31/03/2020	26:36
Male	40	Democratic Republic of Congo	São Paulo	No	Wife and children	Self-employed	2009	Portuguese/ French	31/03/2020	63:07
Male	30	Syria	São Paulo	No	Friends	Freelancer	2017	English	31/03/2020	53:58
Male	30	Democratic Republic of Congo	São Paulo	No	Brothers	Vulnerable (unemployed	2016	Portuguese	01/04/2020	21:23
Male	23	Democratic Republic of Congo	São Paulo	No	Wife and children	Employed	2013	Portuguese	01/04/2020	41:27
Male	25	Democratic Republic of Congo	São Paulo	No	Alone	Vulnerable (unemployed)	2014	Portuguese	01/04/2020	53:19
Male	39	Democratic Republic of Congo	Rio de Janeiro	Yes	Wife, children, mother in law, sister in law	Employed (home office)	2008	Portuguese	02/04/2020	51:51
Female	48	Guyana	São Paulo	Yes	Husband	Freelancer	2011	English	02/04/2020	41:13
Female	36	Democratic Republic of Congo	São Paulo	Yes	Mother, husband, and children	Vulnerable (unemployed)	2013	Portuguese	03/04/2020	31:40
Male	27	Syria	Rio de Janeiro	No	Girlfriend	Employed (home office)	2012	Portuguese	03/04/2020	66:52
Male	40	Democratic Republic of Congo	São Paulo	No	Wife and children	Employed (home office)	2013	Portuguese	03/04/2020	63:01
Male	20	Democratic Republic of Congo	São Paulo	No	Uncle and sister	Employed	2012	Portuguese	04/04/2020	31:54
Male	28	Syria	São Paulo	No	Friends	Freelancer	2015	Portuguese	04/04/2020	26:17

Male	32	Syria	São Paulo	No	Alone	Employed (home office)	2014	Portuguese	04/04/2020	38:19
Female	39	Democratic Republic of Congo	São Paulo	No	Children	Vulnerable (unemployed)	2008	Portuguese	04/04/2020	21:51
Female	37	Venezuela	São Paulo	Yes	Husband, children, and parents in law	Self-employed (food area)	2016	Portuguese	06/04/2020	63:17

Source: Elaborated by the author based on the 29 semi-structured phenomenological interviews with refugees living in the states of São Paulo and Rio de Janeiro between 27/03/2020 and 06/04/2020.
[1] Risk group means if the refugee or a member of his family living in the same house is part of a group with additional risks to COVID-19 (e.g., elderly, people with chronic diseases like health problems and diabetes).
[2] He was employed during the interview. However, he lost his job ten days after that due to his company's cut of costs following the closure of non-essential commerce in Brazil.

Documents of the Study

This section brings a translation from Portuguese to English of the Recruitment text, the study background, and the interview guide with the informed consent Script.

Recruitment text

This text was sent through Whatsapp message and Facebook Messenger

Hello, Good Morning. How are you and your family?

My name is Patrícia Nabuco Martuscelli. I am researcher at the Núcleo de Pesquisa em Relações Internacionais at the Universidade de São Paulo (USP). I am conducting a research on the impact of COVID-19 in refugees in Brazil.

I hope you and your family are safe and well. With this situation of COVID-19, few people have been considering how this pandemic has affected the refugees, their lives, their work. I would like to know if you would like to be part of the research by sharing with me through a Whatsapp call what your perceptions are about that. This conversation will take between 30-40 minutes. I will ask questions about you, how you feel, what you know about this disease, how you are keeping in touch with your families and friends. I can call you at any day and time that will be good for you. My research will focus on how refugees are dealing with the COVID-19 and what your expectations are, considering the answers from the government and the future. Would you like to be part of my research? If you agree, I ask you to answer this message with your Whatsapp number and suggestions of dates and times that you would be available in the next weeks.

Thank you so much for your time and attention. Please feel free to ask me any questions or if you need any further information. You can contact me also by email (patnabuco@alumni.usp.br) or by phone (phone number). I am also sending you the 1-page explanation with further information about my research.

Best Regards,

Patrícia Nabuco Martuscelli

Study Background

Refugees and COVID-19 in Brazil

This is a NUPRI/USP research study about refugees and COVID-19 in Brazil. This research aims to understand refugees' lived experiences during the first weeks of the pandemic in Brazil when the governors of São Paulo and Rio de Janeiro adopted measures to fight the pandemic.

About the Study

We are interested in learning about several issues regarding the experiences of refugees living in Brazil during the COVID-19 pandemic; we want to understand:

- How refugees are dealing with the pandemic, including measures adopted to fight it and how they get information about it;
- How the pandemic has affected their lives and their work situation;
- How the refugees are keeping contact with their friend and families in time of physical distancing;
- How refugees perceive the answers of the government at the federal level, state level, and municipal level;
- What the refugees expect for the future.

Project Team

Patrícia Martuscelli, Ph.D.

Telephone number

patnabuco@alumni.usp.br

Department

NUPRI/USP

Contact Information

For this study, we are conducting interviews using Whatsapp calls to respect the measures of physical distancing. We hope to have consultations with a broad range of refugees living in the states of São Paulo and Rio de Janeiro. The information provided to us will be used in preparing published reports that we hope will generate knowledge to inform public policies and decision-making processes.

For more information about this study, please contact Dr. Patrícia Martuscelli (Principal Investigator) at [telephone number] or patnabuco@alumni.usp.br

Interview Guide with oral informed consent script

Refugees and COVID-19 in Brazil

Introduction – Information about the study:
Thank you very much for agreeing to meet with me. My name is Patrícia Nabuco Martuscelli. I am a researcher from the Núcleo de Pesquisa em Relações Internacionais – Universidade de São Paulo (NUPRI/USP). My research is studying how refugees are affected by COVID-19 in Brazil. This study aims to understand refugees' lived experiences during the first weeks of the pandemic in Brazil when the governors of São Paulo and Rio de Janeiro adopted measures to fight the pandemic. We are interested in learning about several issues regarding the experiences of refugees living in Brazil during the COVID-19 pandemic; we want to understand:

- How refugees are dealing with the pandemic, including measures adopted to fight it and how they get information about it;
- How the pandemic has affected their lives and their work situation;
- How the refugees are keeping contact with their friend and families in time of physical distancing;
- How refugees perceive the answers of the government at the federal level, state level, and municipal level;
- What the refugees expect for the future.

We hope to speak with 20-30 refugees living in the states of São Paulo and Rio de Janeiro. The information provided to us will be used in published reports, academic articles, and blog posts. We may include quotes from those we interview; your name and all information that can identify you will not be recorded or disclosed.

Oral Informed Consent Script (the person will also receive the participation sheet):
Before beginning the interview, I want to emphasize that your participation is voluntary. You are not required to participate, and there is no penalty for not participating. If you consent to be interviewed, you can refuse to answer any question, and you may stop the interview at any time for any reason. You can withdraw from this study at any time, and all the information you gave me will be deleted and not used in the study anymore. Your interview will be stored in secure places following the highest international security standards. No other person will know that you gave me this interview.

We do not expect participation in this interview to benefit you personally. We hope that the project will be of public benefit and generate knowledge to inform public policies and decision-making processes.

This interview is also an opportunity for you to express yourself on an important topic in refugees' lives in Brazil and your life. Perhaps people who have not had the opportunity to have direct contact with refugees will read these research results. You may use this opportunity to inform those who do not know what you think and want, or the difficulties you face, or how you think your life could be better, for example.

I know that you are busy, and I will try to be as focused as possible. I have a series of questions I would like to ask over the next 40 minutes or so. The interview will be audio-recorded unless you prefer that only handwritten notes be taken.

Following the interview, if you have any questions regarding this project, you can contact me, Patrícia Martuscelli, at [telephone number] or patnabuco@alumni.usp.br.

- Do you have any questions about the research project, our research team, or the purpose of this interview? (Yes/No)
- Do you voluntarily agree to participate in the study? (Yes/No)
- Do I have your permission to audio record the interview? (Yes/No)

Thank you. I will now proceed with the interview.

Information on the refugee
- What is your country of origin?
- What is your age?
- In which city do you live? Who lives with you? (e.g., your family, friends. Did your family come after you?)
- When did you arrive in Brazil?

Information on COVID-19
- What do you know about COVID-19?
- How do you get information about this new disease?
- How are you dealing with the COVID-19? (e.g., did you adopt any special measures? Are you in quarantine? Are your children going to school? Since when?)
- Do you have diabetes, heart problems, breathing problems, or any chronic diseases? How about your Family?
- How are you feeling at this moment?

Work
- Do you work? Are you currently employed? (e.g., if yes, what do you do?)
- Is anyone in your house working too?
- How was your work situation before this pandemic? And after?
- What is the situation of your Family at this moment?

Contact with Family and Friends
- When you need help, who usually helps you?
- How are you having contact with your Family?
- How are you having contact with your friends?
- How is the situation of COVID-19 in your country of origin?
- How do you think that this pandemic affects your life?
- How do you think that this pandemic affects the life of refugees in general?

Answers from the Brazilian authorities
I will ask you three questions now that may look the same, but they are different. If you do not want to answer them or do not know, that is not a problem. Just let me know, ok?

- How do you think that the president of Brazil is dealing with the pandemic?
- How do you think that the mayor of your city is dealing with the pandemic?
- How do you think that the governor of your state is dealing with the pandemic?

- How is the government in your country dealing with this pandemic?

Advice and Future thoughts

- How do you think that the Brazilian government could help refugees during this time of COVID-19?
- If you could give the Brazilian government recommendations about this situation of COVID-19, what would you say?
- If you could advise another refugee about this issue of COVID-19, what would you say to him/her?
- What do you expect for the next three months?
- What do you expect in the future in general?
- Would you like to add any information or idea that I did not ask you about and think it is important for this topic of refugees and COVID-19 in Brazil?

End of the interview

I asked you all the questions that I needed to ask. Thank you so much for answering my questions. Your interview contributed a lot to my study. I will send you the results of my research as soon as I finish writing them. At any time, if you do not feel comfortable or if you do not want to be part of this research anymore, you can contact me without any problems. You have my phone number and my email. Please enter in contact with me whenever you need. Thank you again for your time and attention.
